# Retracted: miR-451a Inhibited Cell Proliferation and Enhanced Tamoxifen Sensitive in Breast Cancer via Macrophage Migration Inhibitory Factor

**DOI:** 10.1155/2022/9758629

**Published:** 2022-07-01

**Authors:** BioMed Research International

*BioMed Research International* has retracted the article titled “miR-451a Inhibited Cell Proliferation and Enhanced Tamoxifen Sensitive in Breast Cancer via Macrophage Migration Inhibitory Factor” [1], because several images were found to be duplicated as follows:

- Figure 5(b) LCC2 Lv-miR-451aNc panel is identical to both the Figure 5(b) LCC2 Lv-miR-451a panel and the Figure 8(b) siMIF panel.

- Figure 7(a) MCF7 GAPDH lanes are a rotated version of the Figure 7(a) LCC2 GAPDH lanes.

- Figure 8(a) LCC2 0 h panel is identical to the Figure 8(a) MCF-7 0 h panel.

The authors responded to explain that the errors were introduced during manuscript preparation. However, due to the number of duplicated images the Editorial Board no longer consider the data to be reliable.

The article is therefore retracted with the agreement of the editorial board.

The authors agree with this retraction.
